# Hidden Order and Dimensional Crossover of the Charge Density Waves in TiSe_2_

**DOI:** 10.1038/srep37910

**Published:** 2016-11-29

**Authors:** P. Chen, Y.-H. Chan, X.-Y. Fang, S.-K. Mo, Z. Hussain, A.-V. Fedorov, M. Y. Chou, T.-C. Chiang

**Affiliations:** 1 Department of Physics, University of Illinois at Urbana-Champaign, 1110 West Green Street, Urbana, Illinois 61801-3080, USA; 2 Frederick Seitz Materials Research Laboratory, University of Illinois at Urbana-Champaign, 104 South Goodwin Avenue, Urbana, Illinois 61801-2902, USA; 3 Advanced Light Source, Lawrence Berkeley National Laboratory, Berkeley, California 94720, USA; 4 Institute of Atomic and Molecular Sciences, Academia Sinica, Taipei 10617, Taiwan; 5 School of Physics, Georgia Institute of Technology, Atlanta, GA 30332, USA; 6 Department of Physics, National Taiwan University, Taipei 10617, Taiwan

## Abstract

Charge density wave (CDW) formation, a key physics issue for materials, arises from interactions among electrons and phonons that can also lead to superconductivity and other competing or entangled phases. The prototypical system TiSe_2_, with a particularly simple (2 × 2 × 2) transition and no Kohn anomalies caused by electron-phonon coupling, is a fascinating but unsolved case after decades of research. Our angle-resolved photoemission measurements of the band structure as a function of temperature, aided by first-principles calculations, reveal a hitherto undetected but crucial feature: a (2 × 2) electronic order in each layer sets in at ~232 K before the widely recognized three-dimensional structural order at ~205 K. The dimensional crossover, likely a generic feature of such layered materials, involves renormalization of different band gaps in two stages.

The rich physics of CDW is exemplified by the varied properties of a large number of transition-metal dichalcogenides^1^,^2^,^3^,^4^,^5^,^6^,^7^,^8^,^9^,^10^, which are additionally of interest for their potential as alternates of graphene/graphite for electronic applications. TiSe_2_ is an especially simple case. Its crystal structure (Fig. 1A) consists of Se-Ti-Se trilayers loosely bonded together by van der Waals forces^11^. It undergoes a second-order CDW transition at *T*_*C*_ ~ 205 K to a commensurate (2 × 2 × 2) superlattice^7^,^9^. There is no relevant Fermi surface nesting, and the traditional picture of Peierls’ instability or coupling via Kohn anomalies does not apply, but the nature of the transition remains under intense debate^12^,^13^,^14^,^15^,^16^,^17^. The crystal in the normal state possesses a small indirect gap. Referring to the pictures of the Brillouin zones for both the normal and CDW phases shown in Fig. 1B–D, the top of the valence band, located at the Γ point, is of the Se 4*p* character, and the bottom of the conduction band, located at the L point, is of the Ti 3*d* character. An early theory invoked an excitonic interaction across the gap, which leads to band folding, gap widening, and energy minimization^16^,^18^. A more recent view is that a (2 × 2 × 2) lattice distortion causes the Se 4*p* and the Ti 3*d* states across the gap to couple via the crystal potential of the distorted lattice^3^,^14^,^16^. The mathematical formulation is similar for the two cases, but the underlying physics is different. In the latter case, the transition is purely a band-structure effect, requiring no higher-order electronic interactions; the coupled electron-lattice system evolves, or distorts, to minimize the total energy.

Our systematic three-dimensional band structure mapping of this system using angle-resolved photoemission spectroscopy (ARPES) proves that the above picture focusing on a single (2 × 2 × 2) coupling is incorrect or too simplistic. Aided by first-principles calculations, we show that while the band dispersion is weak along *z* (perpendicular to the layers), its contribution to the total energy difference between the normal and CDW phases is not negligible. A further impetus for our reinvestigation is the recent report of a (2 × 2) CDW transition in single-layer TiSe_2_^19^,^20^, which provided an intriguing clue about the bulk transition. Specifically, a speculation was that the three-dimensional transition might involve a hidden two-dimensional order never considered or detected before. Indeed, our results reveal a number of surprises. A hitherto undetected (2 × 2) two-dimensional electronic order sets in at ~232 K before the known three-dimensional structural transition at ~205 K. The system does not become a direct-gap semiconductor as widely assumed previously based on the (2 × 2 × 2) folding of the conduction band minimum at the L point back to the Γ point. Rather, the system remains an indirect-gap semiconductor because the valence band maximum shifts from Γ to A^*^.

## Results

### Electronic band structure in the normal and CDW phases

**Figure 1:**
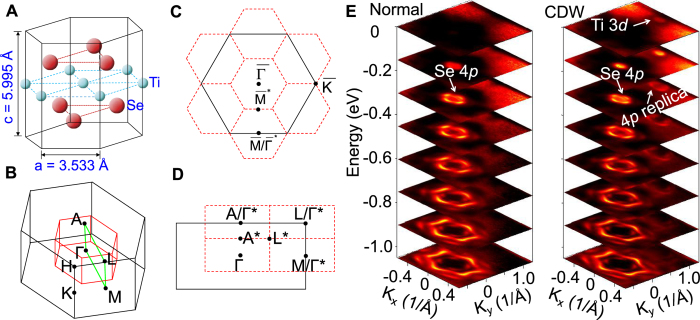
Crystal structure and electronic band structure of TiSe_2_ measured by ARPES. (**A**) Crystal structure of bulk TiSe_2_. (**B**) Three-dimensional Brillouin zones of the (1 × 1 × 1) and (2 × 2 × 2) structures outlined in black and red, respectively. (**C**) Projected two-dimensional Brillouin zones in the basal plane. (**D**) Projected two-dimensional Brillouin zones in a vertical plane. (**E**) ARPES intensity maps at various energies taken with 58 eV photons for the normal phase at 300 K and the CDW phase at 10 K.

A set of ARPES intensity maps at various energies below the Fermi level (Fig. 1E) shows the evolution of the bands, where *k*_*x*_ and *k*_*y*_ point along the 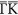 and 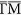 directions, respectively. For the normal phase at 300 K, emission from the Se 4*p* states appears as a circle centered at 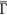 at energies near the Fermi level; it become a hexagon and then a warped hexagon with additional details at energies farther away from the Fermi level. Similar features are evident for the CDW phase at 10 K; additionally, a weak replica by (2 × 2 × 2) folding of the Se 4*p* bands is clearly seen centered at 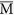 = 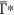 (*k*_*x*_ = 0 and *k*_*y*_ = 1.03 Å^–1^). Furthermore, a tiny circle appears at 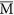 = 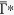 at the Fermi level, which comes from the Ti 3*d* conduction band minimum. Such measurements were repeated for many photon energies in order to systematically change the *k*_*z*_ of the initial state; here, we make use of a free-electron approximation for the final band, with the inner potential set at 13 eV.

### Calculated band structure

**Figure 2:**
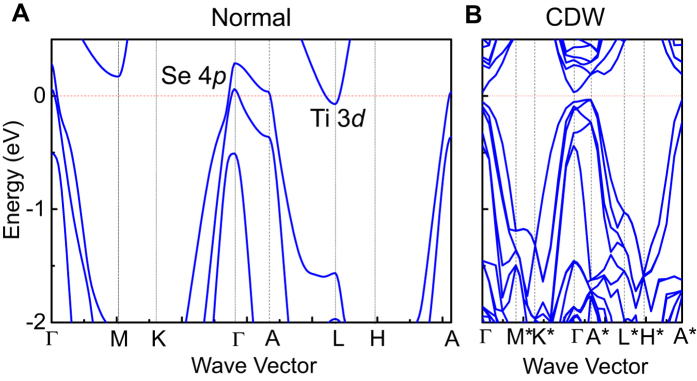
First-principles band structure. Calculated HSE band structure for (**A**) the normal phase and (**B**) the CDW phase.

**Figure 3:**
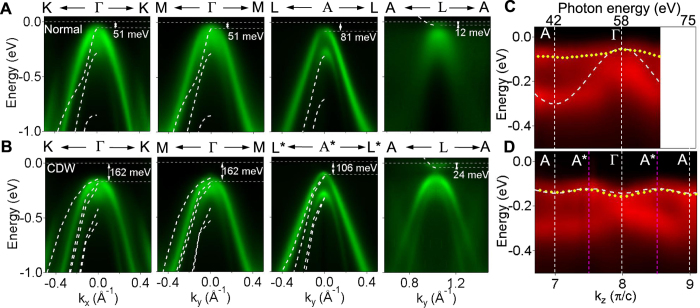
Three dimensional band mapping of TiSe_2_. ARPES spectra for (**A**) the normal phase at 300 K and (**B**) the CDW phase at 10 K along various directions as labeled. Some experimental band edge energies are indicated. The photon energies used were 58, 75, 46, and 67 eV for the data centered about Γ, A, L, and A*, respectively. (**C**) and (**D**) are ARPES intensity maps as a function of *k*_*z*_ or photon energy for the normal and CDW phases, respectively. The yellow dotted curve for each case marks the experimental dispersion relation of the topmost valence band, obtained by fitting to the energy- or momentum-distribution curves. In all cases, the white dashed curves are calculated bands shifted in energy by matching the experimental Ti 3*d* band bottom or Se 4*p* band top where appropriate. The energy shifts for the valence and conductions bands are −345 and 30 meV for the normal phase, and −90 and −80 meV for the CDW phase, respectively.

A calculated HSE band structure is presented in Fig. 2A for the normal phase. The Se 4*p* valence band maximum at Γ and the Ti 3*d* conduction band minimum at L are separated by a negative gap of 0.36 eV, which differs from the experimental value of +0.039 eV (Fig. 3A). This calculation assumes a frozen (1 × 1 × 1) structure at *T* = 0, but this is not a stable or the ground-state structure. Thus, the computed band structure including the gap is just an approximation and should not be expected to accurately describe the experimental results for the (1 × 1 × 1) phase that exists only at *T* > *T*_*C*_. By contrast, there is no such concern for the (2 × 2 × 2) CDW phase at very low temperatures. The calculation (Fig. 2B) shows that the L point is back folded to the zone center, and the Ti 3*d* conduction band minimum is now repeated at the zone center. The CDW interaction pushes the Se 4*p* valence band maximum at Γ to a lower energy, resulting in a positive gap. This energy lowering interaction at Γ leaves the nearby A* point at a higher energy, which becomes the new valence band maximum. The system in the CDW phase remains an indirect-gap semiconductor, contrary to the long-held assumption of a direct-gap semiconductor.

### ARPES maps and perpendicular dispersion

Detailed band mapping results centered about Γ, A, L, and A* along major symmetry directions are presented in Fig. 3A and B for the normal and CDW phases with the sample at 300 and 10 K, respectively. The energies of the valence band maximum and conduction band minimum are marked. The photon energies used were 58, 75, 46, and 67 eV for the data centered about Γ, A, L, and A*, respectively. For comparison, the calculated band dispersion relations are shown as white dashed curves, shifted in energy where appropriate by matching the experimental Ti 3*d* band bottom or the Se 4*p* band top. The experimental conduction band minimum at L/Γ* dips just below the Fermi level in both the normal and CDW phases at nearly the same energy. Also observed is a weak replica of the Se 4*p* valence bands centered about L in the CDW phase due to zone repeating. A fuzzy remnant of the replica bands can be seen for the normal phase, which has been attributed to fluctuation effects^18^ and can be related to the fuzzy x-ray thermal diffuse scattering peaks centered about the superlattice Bragg positions^21^.

Figure 3C and D present ARPES maps of the top valence bands as a function of *k*_*z*_ at *k*_*x*_ = *k*_*y*_ = 0, obtained by scanning the photon energy, for the normal and CDW phases, respectively. The experimental dispersion for the topmost valence band is indicated by a yellow dotted curve in each case; also shown is the theoretical dispersion (white dashed curve). For the CDW phase, the agreement is excellent, including the x2 periodicity. The valence band maximum is indeed at the A* point, and the effective mass at Γ is reversed, as the theory predicts. The theoretical indirect gap is 0.07 eV, in close agreement with the experimental value of 0.082 eV.

### Temperature dependence of the band gap and determination of the transition temperatures

**Figure 4:**
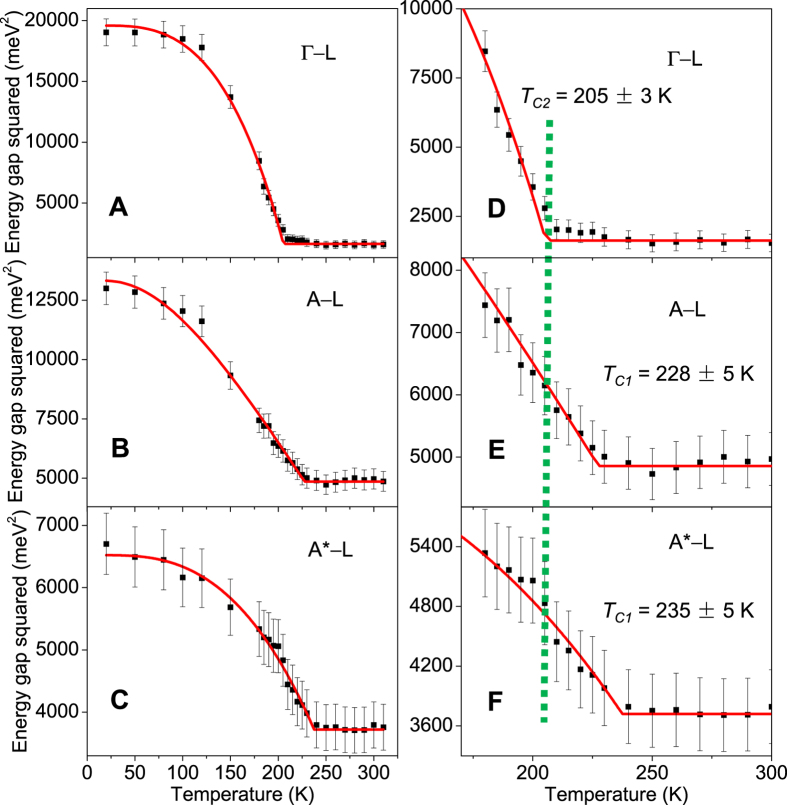
Temperature-dependent band gaps and transition temperatures. The data points represent square of the experimental energy gap between the conduction band bottom at L and the valence band top at (**A**) Γ, (**B**) A, and (**C**) A* as a function of temperature. The energy of each band at each *k* point is determined from fitting to the energy- or momentum-distribution curves. The error bar is deduced from the standard deviation of the fitting. The red curves are fits. (**D**–**F**) present corresponding detailed views near the onset. The transition temperatures *T*_*C*1_ and *T*_*C*2_ deduced from fitting are indicated. The bulk transition at *T*_*C*2_ is indicated by a green vertical dashed line.

Figure 4 shows the experimental temperature dependence of the valence band edge energies at Γ, A, and A* relative to the conduction band minimum at L as the sample is cooled from the normal phase into the CDW phase. Once the CDW phase sets in, points A and L both become Γ*, but the band folding effects are weak for the small lattice distortion, and the parentage of each band remains largely unaffected. Plotted in Fig. 4A–C are squares of the three gap energies, Δ(Γ), Δ(A), and Δ(A*). All three cases show a gap widening transition as the temperature lowers, but the onset temperatures are not the same. Figure 4D–F show zoom-in views near the onsets. The square of the gap energy is expected to follow a mean field behavior for *T* near but below *T*_*C*_:


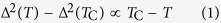


This linear onset is evident in the data and permits a precise determination of the transition temperature. The red curves in Fig. 4 are fits using a semi-empirical BCS-type gap equation^20^, which reduces to a linear function near the onset. Figure 4D shows that the onset of Δ(Γ) is at *T*_*C*2_ ~ 205 K, which agrees well with the known bulk transition temperature. On the other hand, the gaps Δ(A) and Δ(A*) show a substantially higher onset temperature *T*_*C*1_, which agrees closely with the (2 × 2) transition temperature of *T*_*C*0_ = 232 ± 5 K found in single-layer TiSe_2_^20^. The implication is that the CDW transition in bulk TiSe_2_ actually consists of two stages. Upon lowering the temperature, the first transition involves a (2 × 2) ordering of each individual layer at *T*_*C*1_, but the CDW order in neighboring layers is phase uncorrelated. The second transition involves an anti-phase locking of neighboring layers at *T*_*C*2_ to form the (2 × 2 × 2) structure.

## Discussion

An intermediate partially-ordered phase between *T*_*C*1_ and *T*_*C*2_ is entirely consistent with the highly anisotropic nature of the system. Referring to Fig. 3C, the nearly flat experimental dispersion for the top valence band between Γ and A in the normal phase suggests that a (2 × 2 × 2) superlattice modulation connecting Γ and L and a (2 × 2 × 1) superlattice modulation connecting A and L can be both important for driving the CDW transition. In view of the higher transition temperature for the single layer, it is reasonable that bulk TiSe_2_ undergoes (2 × 2) transitions in individual layers first. This transition involves the coupling between A and L, and so the corresponding gap Δ(A) shows an onset at the single-layer transition temperature *T*_*C*1_ = *T*_*C*0_ as seen in experiment. The period doubling along *z* mediated by the van der Waals interlayer coupling is expected to be a weaker effect, and it happens at the lower bulk transition temperature *T*_*C*2_. Because the (2 × 2 × 2) ordering involves the coupling between Γ and L, the gap Δ(Γ) should show an onset at *T*_*C*2_ as seen in Fig. 4D. The data seem to show a hint of a tiny onset at *T*_*C*1_ as well, but it is about the size of the noise. The gap Δ(A*) involves both the (2 × 2 × 1) and (2 × 2 × 2) coupling, and a single-component fit is not necessarily accurate, but the dominant effect should come from the stronger transition at *T*_*C*1_ as seen in Fig. 4F.

Calculations for the two different stacking patterns, (2 × 2 × 1) and (2 × 2 × 2), yield energy lowering per chemical unit of 3 and 4 meV, respectively, relative to the (1 × 1 × 1) structure. The closeness of the two energies suggests that the two-dimensional (2 × 2) distortion is the main driver of the CDW phase. The x2 stacking along *z* lowers the energy a little further, and experimentally it happens at a lower temperature. Theoretical calculations based on energy minimization reveals no energy barriers because the distortion pattern is simple with a monotonic lowering of the energy toward the minimum. The partially ordered intermediate phase between *T*_*C*1_ and *T*_*C*2_ does not necessarily reveal itself in standard diffraction measurements because of the random stacking of the (2 × 2) layers; static long-range structural correlation is needed for superlattice reflection. Indeed, this hidden electronic order has never been detected before by diffraction.

The presence of two transitions associated with two different energy gaps indicates that the CDW transition in TiSe_2_ can be attributed to electron-lattice coupling with a dimensional crossover. A transition, either (2 × 2) within the layers or (2 × 2 × 2) for the three-dimensional organization, leads to band folding, renormalization of the band shapes, and widening of the gaps. The last effect contributes directly to energy lowering of the system. The overall CDW transition is a three-dimensional phenomenon, but it exhibits a strong two-dimensional driver that leads to a separate transition. While the Fermi level lies slightly above the conduction band bottom for both the normal and CDW phases because of n-type self-doping, Fermi surface nesting does not appear to play a role and the system remains a doped indirect-gap semiconductor after the transition. Given the prototypical status of TiSe_2_ within the very large class of CDW transition metal dichalcogenides with similar layered structures, the findings reported herein are likely general and relevant to the development of a comprehensive understanding of the physical behavior of these materials in terms of dimensional effects.

## Methods

### Experimental Details

High quality TiSe_2_ single crystals were synthesized by iodine-vapor transport with excess Se^21^. The samples are semimetallic in agreement with prior studies and our own photoemission measurements. This behavior can be attributed to n-type self-doping of the material caused by Se vacancies. This self-doping effect is common in layered selenides including Bi_2_Se_3_^22^ and SnSe^23^. ARPES measurements were performed at beamlines 12.0.1 and 10.0.1, Advanced Light Sources, Lawrence Berkeley National Laboratory, using samples freshly cleaved *in situ*. The system energy resolution was less than 20 meV, and the angular resolution was 0.2°. The in-plane orientation of the sample was precisely determined from the symmetry of the measured constant-energy ARPES maps. The sample temperature was maintained at 300 and 10 K, respectively, for the normal and CDW phases, except during temperature scans. ARPES Measurements were made using photon energies ranging from 30 to 100 eV in order to systematically vary *k*_*z*_, the momentum component along the surface normal. We assume a free-electron final band dispersion relation shifted by an inner potential, which should work well for the photon energy range used in the experiment^10^,^24^,^25^. The inner potential was set empirically to 13 eV, a typical and reasonable value, in order to place the band extrema at the expected zone center or boundary points.

### Theoretical Calculations

First-principles calculations were performed using the Vienna *ab initio* package (VASP)^26^,^27^,^28^ with the projector augmented wave method^29^,^30^. We included the semi-core 3*p* electrons of Ti in the calculation. With an energy cutoff of 320 eV for the plane wave expansion of the wave functions, the system energy converged to better than 1 meV per chemical unit. A *k*-mesh of 18 × 18 × 18 (9 × 9 × 9) was used in the self-consistent calculations for the normal (CDW) phase. The computed phonons in the normal phase exhibited instabilities near the L and M points, in agreement with previous results^3^. A CDW distortion pattern was constructed by including all symmetry-related soft modes at L, which was then further refined by energy minimization using the generalized gradient approximation (GGA) with the Perdew-Burke-Ernzerhof (PBE) functional^31^. A test calculation based on PBE was performed first for a monolayer TiSe_2_, which resulted in an optimized lattice constant of *a* = 3.538 Å. This value agrees well the experimental value of *a* = 3.534 Å for bulk TiSe_2_. Since PBE is known to have difficulties in predicting the interlayer separation in layered structures with the van der Waals interaction between the layers, the experimental lattice constants *a* = 3.534 Å and *c* = 6.008 Å were used for further optimization of the atomic coordinates for the CDW phase. The final optimized distortion pattern is shown in Fig. S1. It has a lower energy than the normal phase by 4 meV per TiSe_2_ chemical unit. Similar energy lowering was also obtained by using the local density approximation (LDA) with the experimental lattice constants. Our results are consistent with prior reports^3^,^32^.

## Additional Information

**How to cite this article**: Chen, P. *et al.* Hidden Order and Dimensional Crossover of the Charge Density Waves in TiSe_2_. *Sci. Rep.* **6**, 37910; doi: 10.1038/srep37910 (2016).

**Publisher's note:** Springer Nature remains neutral with regard to jurisdictional claims in published maps and institutional affiliations.

## Supplementary Material

Supplementary Information
