# Supplementary material for: Hidden Order and Dimensional Crossover of the Charge Density Waves in TiSe2
Source: Sci Rep. 2016 Nov 29;6:37910. doi: 10.1038/srep37910 (PMC5126568; doi:10.1038/srep37910)
Supplement: Supplementary Information [file srep37910-s1.pdf]

Supplementary Materials

Hidden Order and Dimensional Crossover of the Charge Density Waves in $TiSe_2$

P. Chen,[1,2,3] Y.-H. Chan,[4] X.-Y. Fang,[1,2] S.-K. Mo,[3] Z. Hussain,[3] A.-V. Fedorov,[3] M.Y. Chou,[4,5,6,*] and T.-C. Chiang[1,2,6,*]

[1]Department of Physics, University of Illinois at Urbana-Champaign, 1110 West Green Street, Urbana, Illinois 61801-3080, USA

[2]Frederick Seitz Materials Research Laboratory, University of Illinois at Urbana-Champaign, 104 South Goodwin Avenue, Urbana, Illinois 61801-2902, USA

[3]Advanced Light Source, Lawrence Berkeley National Laboratory, Berkeley, California 94720, USA

[4]Institute of Atomic and Molecular Sciences, Academia Sinica, Taipei 10617, Taiwan

[5]School of Physics, Georgia Institute of Technology, Atlanta, GA 30332, USA

[6]Department of Physics, National Taiwan University, Taipei 10617, Taiwan

* Correspondence to: tcchiang@illinois.edu (T.-C.C.); mychou6@sinica.edu.tw (M.Y.C.)

Additional Details for Theoretical Calculations

The band structure for the optimized structure was calculated using the Heyd-Scuseria-Ernzerhof (HSE) functional including 25% exact exchange [1] on a 12x12x8 (6x6x4) *k*-mesh for the normal (CDW) phase. The band energy at an arbitrary *k* point was deduced by interpolating the Hamiltonian based on maximally localized Wannier functions using the Wannier90 package [2, 3]. Calculations were performed both with and without spin-orbit coupling (SOC) in order to assess the importance of the SOC. All of the results cited in the main text are with the SOC included. The band structures without the SOC are shown in Fig. S2. A comparison of Figs. 2 and S2 reveals that while the SOC causes the bands to split and shift, it does not affect our discussion of the general CDW features. Thus, the SOC does not have a direct impact on the CDW phenomenon in this specific case, perhaps fortuitously, but it is essential for a detailed comparison of calculated and experimental band structure. None of the previous first-principles investigations for the CDW phase of this system included the SOC.

Prior calculations of the band structure of the normal phase using either the LDA or GGA gave a significant negative band gap [4-7]. In our calculation, this negative gap was reduced to 0.36 eV by using the HSE functional with an exact exchange component. Additional quasiparticle calculations using the many-body perturbation method with the GW approximation were also performed for the normal phase using the HSE wave functions as the starting point. The iterative improvement of the Green function G was included at the G4W0 level, which gave a similar band structure with nearly rigid shifts of the valence and conduction bands that reduced the negative gap to 0.18 eV. Since the normal phase exists only above $T_C \sim 205$ K, the calculated band structure for the assumed (1x1x1) structure, with the implicit assumption of $T = 0$ in such

first-principles calculations, is only an approximation and does not account for possible band renormalization by thermal effects.

Question about 2D long-range order

The theorem by Mermin-Wagner precludes long-range order with sufficiently short-ranged interactions when the dimension $d \leq 2$ [8]. However, it has been reported that long-range Coulomb interactions between charges can stabilize the CDW phase [9].

Relationship between energy lowering and gap renormalization

The computed energy lowering associated with CDW formation, 3 and 4 meV each chemical unit for the (2x2x1) and (2x2x2) structures, respectively, is much smaller than the band gap energy change. While the two quantities are related, the numerical values are not directly comparable. Typically, the former is smaller than the latter. Note that the DFT total energy contains more terms beyond the band energy. Furthermore, the band energies of the occupied states change mostly near the gap, with the rest not much affected. The total energy lowering cannot be extracted directly from just the gap renormalization.

The Γ-M gap

The wave vector connecting A and L corresponds to a 2x2x1 modulation, and the wave vector connecting Γ and L corresponds to a 2x2x2 modulation. For both cases, the coupling is between the conduction and valence band states across the gap. A 1x1x2 modulation would couple Γ with A and L with M, but such coupling would not involve states across the gap and therefore could be ignored to first order. The coupling between Γ and M should also be considered for a complete description of the system. An analysis of the Γ-M gap is given in Fig.

S3. The measured temperature dependence of this gap yields a transition temperature of 206 ± 5 K, which agrees with that for the Γ-L gap.

Despite this similarity, it should be emphasized that the coupling relevant to the Γ-M gap is not the same as that for the Γ-L gap. The key point is that the coupling between the conduction band bottom at the M point and the valence top at Γ in the 1x1x1 phase is essentially negligible. This point is illustrated by the calculated HSE band structures shown in Fig. S4 for the 2x2x2 and 2x2x1 phases. The valence band top at Γ gets strongly renormalized to a lower energy when the structure changes from 2x2x1 to 2x2x2 (the two energy positions are indicated in the figure as Γ_{2x2x1} and Γ_{2x2x2}). The conduction band bottom at M in the original 1x1x1 Brillouin zone is folded to the zone center for the 2x2x2 and 2x2x1 phases; its position after folding is labeled M_{2x2x2} and M_{2x2x1}, respectively, and the two energies are nearly identical. This confirms that the conduction band bottom at M is essentially a bystander in the transition. As a result, the renormalization of the Γ-M gap is nearly entirely determined by the renormalization of the valence band top at Γ, which comes from the strong coupling across the Γ-L gap. This explains the similar temperature dependence between the Γ-L and Γ-M gaps, while the relevant coupling is different (strong vs. negligible). The above discussion illustrates an important but perhaps subtle point: *electronic coupling is state-specific, and a correct reciprocal lattice vector does not necessarily imply a strong coupling*. Experimentally, the band energy difference between M and L in the normal phase is smaller than that from calculation. The discrepancy can be attributed to the error in the (1x1x1) calculation as noted above. Evidently, this does not change the general picture outlined above that the coupling across the Γ-M gap is negligible.

A similar analysis can be made for the valence band top at A*, which stays at nearly the same energy for the 2x2x1 to 2x2x2 transition. Thus, the top valence states at and near Γ are strongly involved in the 2x2x2 formation, while the top valence states at and near A* are largely bystanders. The strong renormalization at Γ pushes the original valence band top at this point to a lower energy than that at A*, and the valence band maximum shifts to A* for the 2x2x2 phase.

Fig. S1. Top view of the distortion pattern for the (2x2x2) CDW phase in (A) the first and (B) the second $TiSe_2$ layer. The labels Se1 and Se2 indicate the Se atoms above and below the Ti plane, respectively. The length of each arrow indicates the atomic displacement magnified by a factor of 33. The atomic displacements in two adjacent $TiSe_2$ layers are related by a phase shift of π.

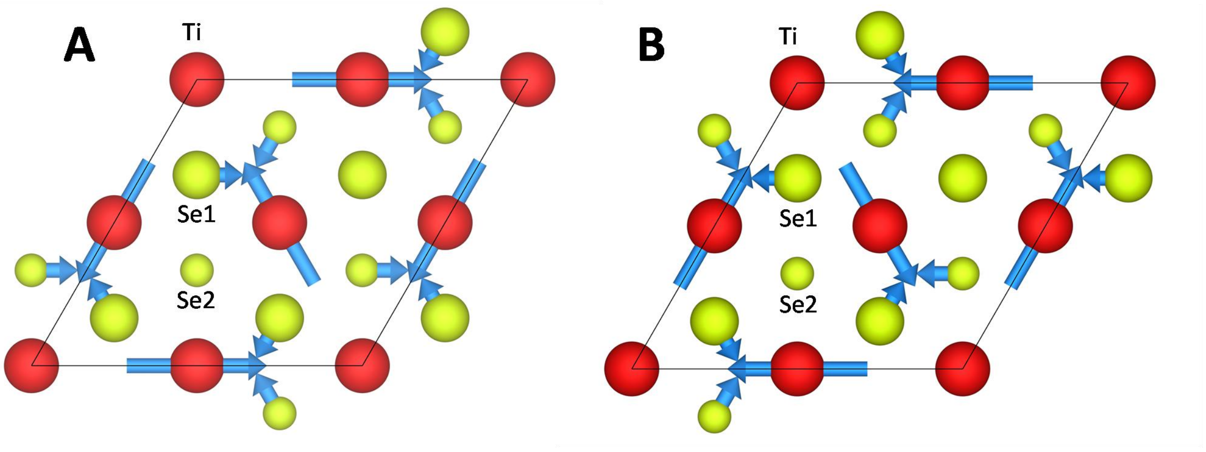

Fig. S2. Calculated HSE band structure with SOC excluded for (**A**) the normal phase and (**B**) the CDW phase. The corresponding results including SOC are shown in Fig. 2.

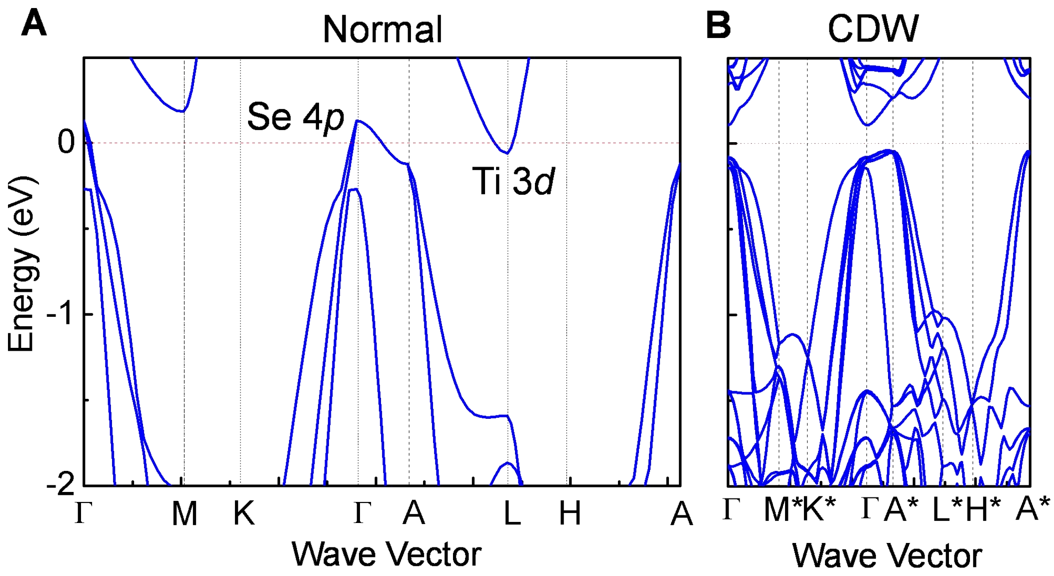

Fig. S3. Measured temperature dependence of various band edge energies and the Γ-M gap.

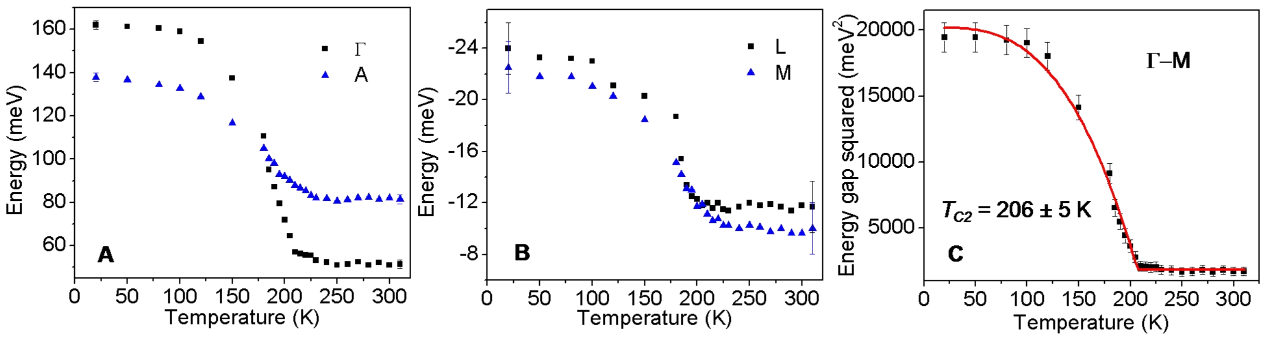

Fig. S4. Calculated band structures for the 2x2x2 and 2x2x1 phases. The valence band top at Γ is labeled Γ_{2x2x2} and Γ_{2x2x1}, respectively, for the two phases, and there is a large energy difference between the two cases. The valence band top at A* is similarly labeled, and the energy difference between the two cases is very small. The conduction band bottom at M in the original 1x1x1 Brillouin zone is folded to the zone center for the 2x2x2 and 2x2x1 phases, and its position after folding is labeled M_{2x2x2} and M_{2x2x1}, respectively; their energies are nearly identical.

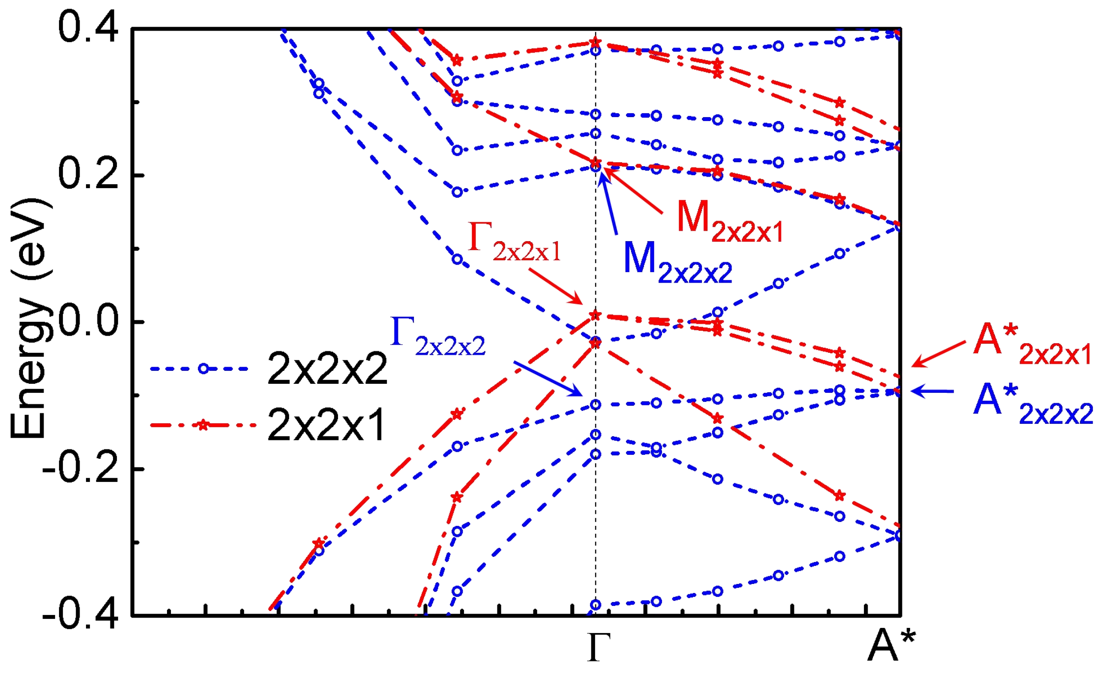

References

1. Heyd, J., Scuseria,G. E. & Ernzerhof, M. Hybrid functionals based on a screened Coulomb potential. *J. Chem. Phys*. **118**, 8207-8215 (2003).

2. Souza, I., Marzari, N. & Vanderbilt, D. localized Wannier functions for entangled energy bands. *Phys. Rev. B* **65**, 035109 (2001).

3. Mostofi, A. A., Yates, J. R., Lee, Y.-S., Souza, I., Vanderbilt, D. & Marzari, N. Wannier90: A tool for obtaining maximally-localised Wannier functions. *Comput. Phys. Commun.* **178**, 685-699 (2008).

4. Calandra, M. & Mauri, F. Charge-density wave and superconducting dome in $TiSe_2$ from electron-phonon interaction. *Phys. Rev. Lett.* **106**, 196406 (2011).

5. Bianco, R., Calandra, M. & Mauri, F. Electronic and vibrational properties of $TiSe_2$ in the charge-density-wave phase from first principles. *Phys. Rev. B* **92**, 094107 (2015).

6. Vydrova, Z. *et al.*, Three-dimensional momentum-resolved electronic structure of 1*T*-$TiSe_2$: A combined soft-x-ray photoemission and density functional theory study. *Phys. Rev. B* **91**, 235129 (2015).

7. Jishi, R. A. & Alyahyaei, H. M. Electronic structure of superconducting copper intercalated transition metal dichalcogenides: First-principles calculations. *Phys. Rev. B* **78**, 144516 (2008).

8. Mermin, N. & Wagner, H. Absence of Ferromagnetism or Antiferromagnetism in One- or Two-Dimensional Isotropic Heisenberg Models. *Phys. Rev. Lett.* **22**, 1133 (1966).

9. Aseev, P. P. & Artemenko, S. N. Stabilization of the surface CDW order parameter by long-

range Coulomb interaction. *Physica B* **407**, 1835-1838 (2012).
